# *In vitro* activity of novel antifungals, natamycin, and terbinafine against *Fusarium*

**DOI:** 10.1128/aac.01913-24

**Published:** 2025-05-15

**Authors:** Enrico Garbe, Ahsan Ullah, Alexander Maximilian Aldejohann, Oliver Kurzai, Slavica Janevska, Grit Walther

**Affiliations:** 1 (Epi-)Genetic Regulation of Fungal Virulence, Leibniz Institute for Natural Product Research and Infection Biology-Hans Knöll Institute 28406, Jena, Germany; 2 Institute for Hygiene and Microbiology, University of Würzburg 9190, Würzburg, Germany; 3 National Reference Center for Invasive Fungal Infections (NRZMyk), Leibniz Institute for Natural Product Research and Infection Biology-Hans Knöll Institute 28406, Jena, Germany; University Children's Hospital Münster, Münster, Germany

**Keywords:** antifungal susceptibility testing, fungal diversity, *Fusarium*, manogepix, olorofim, natamycin, terbinafine, rezafungin, ibrexafungerp

## Abstract

The genus *Fusarium* includes several trans-kingdom pathogens, infecting plants, animals, and humans, with widespread occurrence. Invasive *Fusarium* infections are often destructive due to their prevalence in critically ill patients. Furthermore, severe superficial infections, like keratitis or endophthalmitis with potential loss of vision, are rising in incidence globally. Infections are difficult to treat due to the intrinsic resistance of *Fusarium* against echinocandins and often high minimal inhibitory concentrations (MICs) for azoles. In this article, we assessed the *in vitro* activity of the novel antifungals olorofim, manogepix, rezafungin, and ibrexafungerp, as well as the established but nonstandard drugs, natamycin and terbinafine, against a large set of molecularly identified clinical *Fusarium* isolates via EUCAST microdilution. The tested isolates represent the clinically most relevant species of the *F. solani* (FSSC), *F. oxysporum* (FOSC), *F. fujikuroi* (FFSC), *F. incarnatum-equiseti* (FIESC), *F. dimerum* (FDSC), and *F. redolens* (FRSC) species complexes. While rezafungin and ibrexafungerp showed no effect on the *Fusarium* spp. tested, a general antifungal susceptibility of *Fusarium* against natamycin and manogepix was found. Specific profiles were detailed for terbinafine and olorofim. While species from the FDSC, FSSC, and FIESC show high MICs for olorofim, specific susceptibility rates were found for species of the FFSC and FOSC. Furthermore, we report specific susceptibilities for terbinafine against FDSC, FFSC, and FOSC species. These findings of complex- and species-specific olorofim and terbinafine *in vitro* susceptibilities highlight the need for diagnostics below genus level.

## INTRODUCTION

Fungi from the genus *Fusarium* are ubiquitously present in the environment and do not only include major plant pathogens, but also opportunistic species that can infect humans (1, 2). Infections caused by *Fusarium* are diverse. While superficial infections of skin, nails, or the eye usually affect immunocompetent patients and show a globally rising incidence, systemic fusariosis is a relatively rare devastating disease occurring in immunocompromised or otherwise critically ill patients (3–5). In any case, infections are difficult to treat due to the intrinsic resistance of *Fusarium* species against echinocandins, their limited azole susceptibility, and a rather uncertain clinical response due to low tissue penetration, especially considering eye infections. In addition, correct diagnosis is often delayed and unfortunately confirmed at late infection stages (6–8). Consequently, *Fusarium* was ranked in the high priority group of the World Health Organization's fungal priority pathogens list (9).

*Fusarium* represents a highly diverse genus. Its taxonomy is under permanent revision, with new species being constantly described and species, as well as genus concepts, under debate (10–12). Within the genus, closely related and phenotypically similar species are commonly grouped into species complexes. Of the human pathogenic species, the large majority belongs to the *F. solani* (FSSC), *F. oxysporum* (FOSC), and *F. fujikuroi* (FFSC) species complexes, while species from the *F. incarnatum-equiseti* (FIESC) and *F. dimerum* (FDSC) species complexes are less frequently isolated from clinical sources (13). Within the last years, taxonomic revisions of the clinically relevant FSSC, FOSC, and the FFSC resulted in closer species concepts and numerous new species with a varying pathogenic potential (14–16). Additionally, previous studies reported differences in *in vitro* antifungal susceptibility among *Fusarium* species complexes and species (17–24). Yet, these studies mostly relied on *Fusarium* isolates differentiated only to the species complex level or included only a small proportion of clinically relevant species. Therefore, our aim was to provide antifungal susceptibility data for all clinically relevant *Fusarium* species using a comprehensive panel of clinically retrieved isolates applying the new species concepts. Given the availability of new antifungal drugs like manogepix, olorofim, rezafungin, and ibrexafungerp, we assessed the *in vitro* efficacy for all substances to dissect their behavior on species and species complex level to detail their potential applicability in antifungal therapy. Due to the relevance for the treatment of onychomycosis and fungal keratitis, we further assessed the specific susceptibility of *Fusarium* species for the established, but less studied drugs, natamycin and terbinafine.

Olorofim is a first-in-class substance selectively inhibiting the fungal dihydroorotate dehydrogenase and, in consequence, pyrimidine synthesis (25). It possesses broad *in vitro* activity against a variety of filamentous fungal genera, including *Fusarium* species (19, 26). Manogepix, the active moiety of the prodrug fosmanogepix, is another first-in-class drug targeting the inositol acetyltransferase Gwt1, a key enzyme in the synthesis of GPI-anchored cell wall proteins (27). Similar to olorofim, it shows broad-spectrum activity against clinically relevant fungal genera, including *Fusarium* species (21, 23). Rezafungin is a next-generation echinocandin with prolonged half-life and high plasma concentrations (28). Like other echinocandins, its antifungal activity is carried out by inhibition of the β-1,3-glucan synthase and thus presumably inactive against *Fusarium* (29). Acting on the same target enzyme is ibrexafungerp, a first-in-class triterpenoid antifungal with oral administration (30, 31). While ibrexafungerp shows *in vitro* activity against *Aspergillus* and *Candida* species, no activity was observed against *Fusarium* (32–34). Terbinafine is an allylamine antifungal, which blocks ergosterol synthesis by inhibition of the squalene epoxidase and is mainly used for the treatment of dermatophytes and onychomycosis, while its systemic activity is under debate (35). It also shows species complex-specific *in vitro* activity against *Fusarium* (7, 17). Natamycin is a polyene, which, similar to amphotericin B, inhibits fungal growth by binding to ergosterol (36). Previous studies on a limited number of *Fusarium* isolates showed general *in vitro* activity, which is of special interest for the treatment of fungal keratitis, where natamycin is commonly used (22, 37, 38).

Overall, we found a general *in vitro* activity against all *Fusarium* species tested for natamycin and manogepix but species complex-specific and partially even species-specific antifungal susceptibility for olorofim and terbinafine.

## RESULTS AND DISCUSSION

### Studied strains

**Fig 1:**
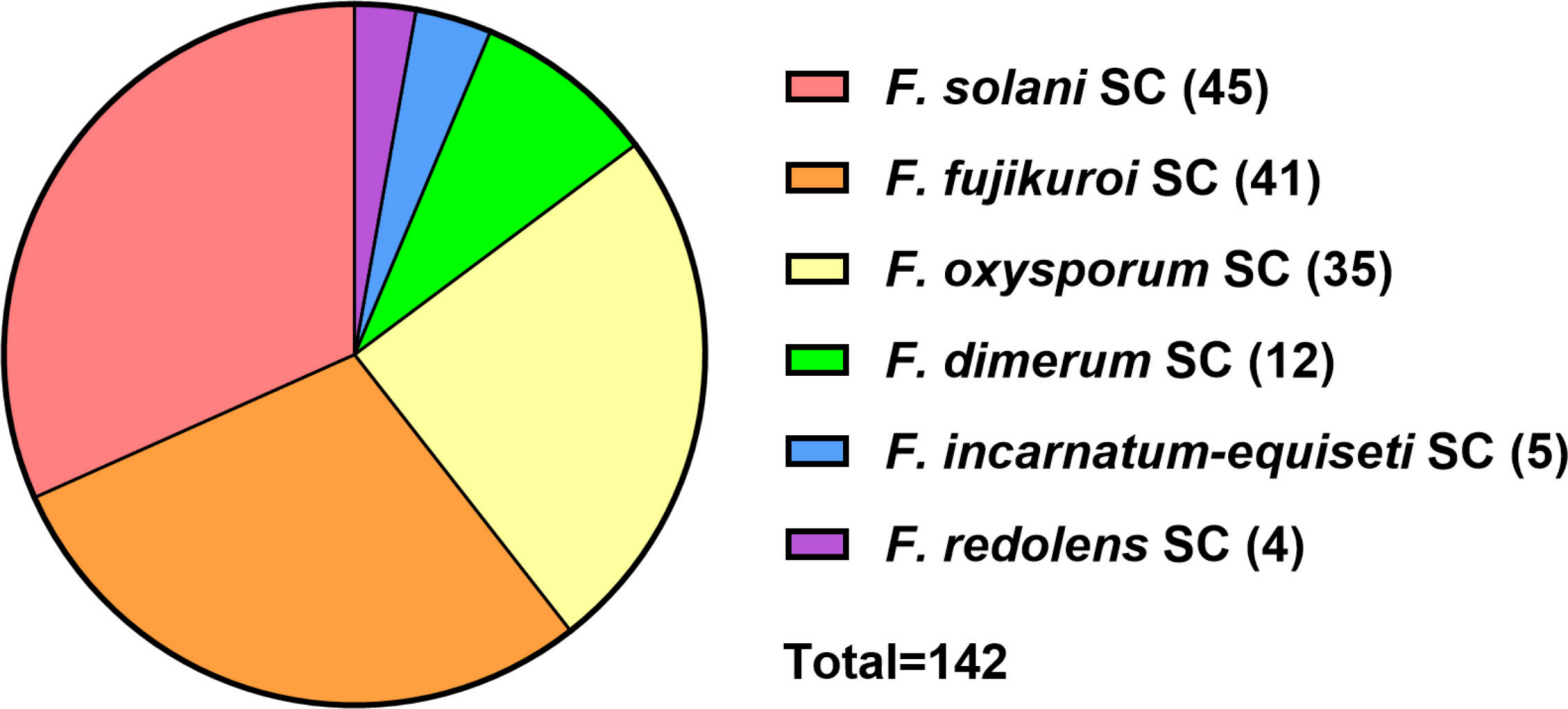
Strains included in this study. Overview of the strains included in this work grouped by their respective species complexes (SC).

The 142 *Fusarium* strains included in this study were chosen to represent the spectrum of clinically relevant *Fusarium* species received by the German National Reference Center for Invasive Fungal Infections (NRZMyk) between 2015 and 2024 (Table S1). All have been retrieved directly from patients or patient-associated material. Species were identified by sequences of the translation elongation factor 1α (*TEF-1 alpha*) and/or the second largest subunit of the RNA polymerase II (*RPB2*). We identified species belonging to six species complexes — FSSC, FOSC, FFSC, FDSC, FIESC, and *F. redolens* species complex (FRSC) (Fig. 1).

### Species identification

By using *TEF* or *RPB2* sequences, 139 strains could be unambiguously assigned to 24 *Fusarium* species (Table 1) . A few isolates that differed by several base pairs from known species without a clear hit, and of which only a single isolate per sequence type was found, were excluded from the study. In three cases, we included isolates that are not identified to the species level: the isolates NRZ-2017–0207, NRZ-2019–0558, and NRZ-2023–1306 are listed as “*F. contaminatum/pharetrum*” because their TEF sequences differ from those of the ex-type strains of *F. contaminatum* and *F. pharetrum* by the same number of nucleotides (one or two bps).

**TABLE 1:** Antifungal susceptibility profiles of *Fusarium* strains included in this study^*a*^^,^^*b*^

Species complex/Species	MIC (mg/L)																MEC (mg/L)			
	Natamycin				Terbinafine				Olorofim (visual assessment)				Olorofim (90% growth inhibition)				Manogepix			
	Range	GM	MIC50	MIC90	Range	GM	MIC50	MIC90	Range	GM	MIC50	MIC90	Range	GM	MIC50	MIC90	Range	GM	MIC50	MIC90
**FDSC (12)**	**2–4**	**2.670**	**2**	**4**	**0.25–4**	**1**	**1**	**2**	**>8**	-^*c*^	-	-	**>8**	-	-	-	**≤0.016**	-	-	-
*F. delphinoides* (2)	2–4	2.828	-	-	1	-	-	-	>8	-	-	-	>8	-	-	-	≤0.016	-	-	-
*F. dimerum* (10)	2–4	2.639	2	4	0.25–4	1	1	2	>8	-	-	-	>8	-	-	-	≤0.016	-	-	-
**FFSC (41)**	**4–8**	**4.503**	**4**	**8**	**0.125–4**	**1.451**	**2**	**2**	**≤0.016–1**	**0.170**	**0.25**	**1**	**≤0.016–1**	**0.073**	**0.06**	**0.25**	**≤0.016–0.03**	**0.016**	**≤0.016**	**≤0.016**
*F. annulatum* (13)	4–8	5.222	4	8	0.125–4	1.532	2	4	≤0.016–0.06	0.022	≤0.016	0.06	≤0.016–0.06	0.018	≤0.016	≤0.016	≤0.016	-	-	-
*F. jonfreemaniae* (6)	4	-	-	-	1–4	1.782	-	-	0.5–1	0.707	-	-	0.25–1	0.445	-	-	≤0.016	-	-	-
*F. musae* (12)	4	-	-	-	1–2	1.189	1	2	0.125–1	0.420	0.5	1	0.03–0.25	0.124	0.125	0.25	≤0.016	-	-	-
*F. verticillioides* (10)	4–8	4.595	4	8	1–2	1.516	2	2	0.125–1	0.354	0.5	1	0.03–0.5	0.081	0.6	0.125	≤0.016–0.03	0.017	≤0.016	≤0.016
**FIESC (5)**	**2–4**	**3.482**	-	-	**32 - > 32**	**55.715**	-	-	**>8**	-	-	-	**0.125 - > 8**	**6.063**	-	-	**≤0.016–0.03**	**0.018**	-	-
*F. clavum* (2)	2–4	2.828	-	-	32 - > 32	45.255	-	-	>8	-	-	-	0.125 - > 8	1.414	-	-	≤0.016	-	-	-
*F. equiseti* (2)	4	-	-	-	>32	-	-	-	>8	-	-	-	>8	-	-	-	≤0.016–0.03	0.022	-	-
*F. flagelliforme* (1)	4	-	-	-	>32	-	-	-	>8	-	-	-	>8	-	-	-	≤0.016	-	-	-
**FOSC (35)**	**2–8**	**4.595**	**4**	8	**0.5 - > 32**	**11.202**	**8**	**>32**	**0.06–1**	**0.329**	**0.5**	**1**	**0.03–1**	**0.164**	**0.125**	**0.5**	**≤0.016–0.06**	**0.017**	**≤0.016**	**≤0.016**
*F. contaminatum* (2)	4	-	-	-	4 - > 32	16.000	-	-	0.125–1	0.354	-	-	0.125–1	0.354	-	-	≤0.016	-	-	-
*F. contaminatum/* *pharetrum* (3)	4	-	-	-	16 - > 32	40.317	-	-	0.06–0.25	0.123	-	-	0.03–0.125	0.061			≤0.016	-	-	-
*F. coriorum* (2)	4–8	5.657	-	-	1–2	1.414	-	-	0.06–0.125	0.087	-	-	0.06	-	-	-	≤0.016	-	-	-
*F. curvatum* (3)	4–8	5.040	-	-	0.5–32	4	-	-	0.25–0.5	0.315	-	-	0.06–0.125	0.098	-	-	≤0.016	-	-	-
*F. languescens* (1)	4	-	-	-	>32	-	-	-	0.25	-	-	-	0.25	-	-	-	0.06	-	-	-
*F. nirenbergiae* (11)	4–8	4.832	4	8	1 - > 32	17.041	>32	>32	0.25–1	0.441	0.5	0.5	0.125–0.5	0.182	0.125	0.25	≤0.016–0.03	0.017	≤0.016	≤0.016
*F. oxysporum* (1)	8	-	-	-	>32	-	-	-	1	-	-	-	0.5	-	-	-	≤0.016	-	-	-
*F. veterinarium* (12)	2–8	4.238	4	8	1 - > 32	7.127	4	>32	0.125–1	0.375	0.5	1	0.125–1	0.198	0.125	0.25	≤0.016–003	0.017	≤0.016	≤0.016
**FRSC (4)**	**4–8**	**4.757**	-	-	**2–4**	**2.378**	-	-	**0.25–0.5**	**0.354**	-	-	**0.06–0.125**	**0.104**	-	-	**≤0.016**	-	-	-
*F. redolens* (4)	4–8	4.757	-	-	2–4	2.378	-	-	0.25–0.5	0.354	-	-	0.06–0.125	0.104	-	-	≤0.016	-	-	-
**FSSC (45)**	**1–16**	**5.443**	**4**	**8**	**8 - > 32**	**60.176**	**>32**	**>32**	**>8**	-	-	-	**2 - > 8**	**14.365**	**>8**	**>8**	**≤0.016–0.06**	**0.016**	**≤0.016**	**≤0.016**
*F. breviconum* (1)	8	-	-	-	>32	-	-	-	>8	-	-	-	>8	-	-	-	≤0.016	-	-	-
*F. falciforme* (8)	4–8	6.619	-	-	>32	-	-	-	>8	-	-	-	4 - > 8	13.454	-	-	≤0.016	-	-	-
*F. keratoplasticum* (10)	1–8	3.732	4	4	>32	-	-	-	>8	-	-	-	>8	-	-	-	≤0.016	-	-	-
*F. metavorans* (1)	8	-	-	-	>32	-	-	-	>8	-	-	-	>8	-	-	-	≤0.016	-	-	-
*F. petroliphilum* (13)	4–8	5.508	4	8	>32	-	-	-	>8	-	-	-	2 - > 8	13.635	>8	>8	≤0.016	-	-	-
*F. solani* (10)	2–16	6.498	8	8	8 - > 32	48.503	>32	>32	>8	-	-	-	>8	-	-	-	≤0.016–0.06	0.018	≤0.016	≤0.016
*F. tonkinense* (2)	4–8	5.657	-	-	>32	-	-	-	>8	-	-	-	4 - > 8	8	-	-	≤0.016	-	-	-

^*a*^ Indicated are the MIC values from visual readout for natamycin, terbinafine and olorofim for all strains included in this study grouped by species complexes and individual species. For olorofim additionally the MIC value for 90% growth inhibition based on spectrophotometric readout is given. For manogepix the MEC value is provided. For all antifungals the range of MIC/MEC as well as the geometric mean (GM) is indicated and if at least ten isolates have been tested additionally MIC50 (50th percentile) and MIC90 (90th percentile).

^*b*^ Bold values refer to the MIC/MEC data for the indicated species complexes.

^*c*^ "-" is shown whenever 10 isolates for the indicated species/species complex have not been tested and thus the calculation of 50th/90th percentile was not possible or when all tested isolates had the same MIC/MEC values.

### Natamycin

For natamycin, a general effectiveness against the tested *Fusarium* species was observed with MICs usually ranging between 2 and 8 mg/L (Table 1). Only one *F. keratoplasticum* and one *F. solani* isolate fell outside this range with MICs of 1 and 16 mg/L, respectively. Generally, for FDSC and FIESC species, lower geometric mean (GM) MICs were observed with 2.67 and 3.482 mg/L, while FSSC species (GM MIC 5.443 mg/L) showed higher values, in particular, *F. falciforme* and *F. solani*. Notably, in contrast to this trend, *F. keratoplasticum* had a lower GM MIC of 3.732 mg/L.

These findings are well in line with the MICs of natamycin against *Fusarium* species previously reported by us and other groups (17, 37, 38). It also confirms the general usability of natamycin in the topical treatment of *Fusarium* eye infections since isolates with natamycin MICs <16 mg/L are considered susceptible (37).

### Terbinafine

FIESC and FSSC species showed consistently high MICs against terbinafine >32 mg/L, with only three isolates below this value — one *F. clavum* isolate with 32 mg/L and two *F. solani* isolates with 8 and 32 mg/L, respectively. In contrast, terbinafine showed comparable *in vitro* activity against all tested species from the FDSC, FFSC, and FRSC, with MICs ranging from 0.125 to 4 mg/L (Table 1). From those three complexes, *F. redolens* showed the highest GM MIC of 2.378 mg/L, while the FDSC and FFSC species were all <2 mg/L. The FOSC species showed varying GM MICs, as well as a notable intraspecies variability of terbinafine susceptibility. Exemplary, *F. nirenbergiae* showed a GM MIC of 17.041 mg/L, while *F. veterinarium* had 7.127 mg/L. Yet, for both species, a MIC range from 1 to >32 mg/L was noted. These findings, based on a larger number of strains tested, discard our previous notion of high terbinafine MICs as a distinction of the FSSC and absent in the FOSC (17). Similar to our findings, Alastruey-Izquierdo et al. reported fluctuating terbinafine MICs for 14 tested FOSC isolates, with MICs ranging from 0.5 to 32 mg/L (7). However, we noticed that the assessment of terbinafine activity can be difficult due to slowly decreasing turbidity and therefore concluded that spectrophotometric MIC assessment might be a potential alternative option.

### Manogepix

The antifungal activity of manogepix against *Fusarium* and other filamentous fungi is usually assessed as the minimal effective concentration (MEC) comparable to the assessment of echinocandin activity (21, 24, 39). Although the mycelium clouds the wells nearly as in the negative control, manogepix leads to abnormal hyphal growth resulting in a flat phenotype on the bottom of the well of the microdilution plate (Fig. S1). Furthermore, the mycelium density for particular species (e.g., *F. dimerum*) was reduced, and spore formation was absent in most strains. Using these criteria, all tested *Fusarium* isolates showed manogepix susceptibility with MECs usually ≤0.016 mg/L, except for six isolates showing MECs of 0.03 or 0.06 mg/L (Table 1) . No taxon-specific susceptibility was detected.

This finding of a general *Fusarium* susceptibility against manogepix confirms previous studies using MEC readout for multiple isolates from the FOSC and FSSC and a smaller quantity of the FDSC, FFSC, and FIESC isolates, with MECs ranging from ≤0.015 to 0.06 mg/L (21, 24, 39). Notably, no differences using either Clinical and Laboratory Standards Institute (CLSI) or EUCAST methods were reported, except for one study by Rivero-Menendez and colleagues, where CLSI testing found more *Fusarium* strains to be susceptible compared to EUCAST methods (40).

In summary, the general *in vitro* susceptibility of *Fusarium* renders manogepix as a promising candidate for future therapeutic approaches of these otherwise difficult-to-treat fungi. *In vivo* studies in mice already reported the successful treatment of disseminated fusariosis with manogepix in effective concentrations of 0.015 to 0.03 mg/L, which fits the *in vitro* observations (41, 42). Additionally, the first case reports already describe the successful use of manogepix for the treatment of *Fusarium* infections (43, 44).

### Olorofim

For olorofim, we found considerable differences in susceptibility between the individual species complexes and partially, even at species level. The FDSC, FIESC, and FSSC species all displayed resistance against olorofim with a MIC >8 mg/L (Table 1). In contrast, isolates of the FFSC, FOSC, and FRSC were susceptible with GM MICs of 0.17, 0.329, and 0.354 mg/L, respectively. For the FFSC, species-specific susceptibility patterns were also visible. While *F. musae* and *F. verticillioides* had similar GM MICs with 0.42 and 0.354 mg/L, respectively, *F. joanfreemaniae* showed a comparably high GM MIC for this complex with 0.707 mg/L. Lastly, *F. annulatum* isolates displayed pronounced olorofim susceptibility of mostly ≤0.016 mg/L and with a GM MIC of 0.022 mg/L.

During our assessment of the olorofim activity against *Fusarium*, we noticed that a visual readout can be difficult due to slow decreasing *Fusarium* growth and thus indistinctive media turbidity in the single wells — potentially resulting in error-prone MIC determination by the experimenter (Fig. S2). Therefore, we also compared visual and spectrophotometric readouts and found overall lower and less scattered MICs for olorofim using a spectrophotometric readout of 90% growth inhibition (Table 1; Fig. S3). However, the classification of isolates from the FDSC, FIESC, and FSSC as non-susceptible generally did not change, as only four isolates had a MIC_90_ <8 mg/L — one each from *F. clavum*, *F. petroliphilum*, *F. tonkinense*, and *F. falciforme*.

These susceptibility profiles are generally consistent with previously performed testing. One study, including one *F. dimerum*, one *F. annulatum* (listed as *F. proliferatum*), and eight FSSC isolates, reported high MICs for *F. dimerum* and FSSC >1 and 0.016 mg/L for *F. annulatum* (18). Another study by Badali et al. investigating olorofim *in vitro* activity against clinical FOSC and FSSC isolates confirmed high MICs for the FSSC (20). They also identified a subset of their FOSC species as either *F. veterinarium* or *F. nirenbergiae* and reported specific GM MICs of 0.391 and 1.19 mg/L, respectively. Yet, in our setting, we found for both species GM MICs very similar to the one reported for *F. veterinarium*. We also observed notably lower intraspecies variability for both species. A third study also reported olorofim susceptibility for FOSC and high MICs for FDSC and FSSC and a MIC of 0.5 mg/L for the sole tested *F. verticillioides* isolate (45). Lastly, using a readout of 50% growth inhibition, olorofim susceptibility has been reported for the FSSC (18, 20). However, since no data of *Fusarium in vivo* susceptibility against olorofim are currently available, the therapeutic relevance of this observation is unclear and requires further investigation.

### Ibrexafungerp and rezafungin

Ibrexafungerp and rezafungin both target the β-1,3-glucan synthase like established echinocandins, for which *Fusarium* resistance is well known (6, 46). Therefore, only a subset of the strains included in this study, representing the clinically relevant species complexes, was tested. For all tested strains, MECs >8 mg/L for both substances were noted, with the exception of one *F. dimerum* strain, which had a MEC of 8 mg/L for rezafungin (Table 2). This finding verifies a previous study showing *Fusarium* resistance against ibrexafungerp (34). The generally high MECs observed for rezafungin are further well in line with the intrinsic resistance of *Fusarium* against other echinocandins like caspofungin (47, 48).

**TABLE 2:** Ibrexafungerp and rezafungin MECs of selected *Fusarium* isolates^*a*^

Species complex/species	MEC (mg/L)	
	Rezafungin	Ibrexafungerp
FDSC		
*F. dimerum* (2021–0751)	8	>8
FFSC		
*F. verticillioides* (2023–0449)	>8	>8
*F. annulatum* (2021–0087)	>8	>8
FIESC		
*F. clavum* (2023–0484)	>8	>8
FOSC		
*F. veterinarium* (2022–0954)	>8	>8
FRSC		
*F. redolens* (2020–515)	>8	>8
FSSC		
*F. solani* (2023–1077)	>8	>8

^*a*^ Shown are the MECs for rezafungin and ibrexafungerp for the indicated *Fusarium* strains representing the clinically relevant species complexes.

### Conclusion

Systemic and superficial *Fusarium* infections represent a serious medical threat due to often delayed diagnosis and limited therapeutic options among the currently available antimycotics. Therefore, we detailed in this study the susceptibility of clinically relevant *Fusarium* species against the novel antimycotics manogepix, olorofim, rezafungin, and ibrexafungerp, and the nonstandard drugs, terbinafine and natamycin, with the latter being especially relevant for the treatment of *Fusarium* eye infections. Thereby, we found distinct susceptibility patterns for terbinafine and olorofim, emphasizing the importance of specific diagnostics of the *Fusarium* species causing the particular infections in order to initiate appropriate therapy. Yet, it needs to be noticed that only a small number of strains for rare opportunistic pathogens like *F. tonkinense* or *F. delphinoides* could be tested, and thus potential diversity within those species and corresponding species complexes could remain unnoticed. Also, the underlying mechanisms for the observed differences in susceptibility of *Fusarium* against the investigated antifungals remain unknown and will require further investigation. The finding of a general *in vitro* susceptibility of all tested *Fusarium* species against manogepix and the effectiveness of olorofim against the FFSC and FOSC were especially encouraging. Although attention should be laid on the investigation of potential resistance phenotypes of *Fusarium* against manogepix and olorofim, like already described for *Candida* and *Aspergillus*, these findings hold promise for the future therapy of these otherwise difficult-to-treat fungi (49, 50).

## MATERIAL AND METHODS

### Strains included in this study

All *Fusarium* strains tested in this study have been received by the NRZMyk from medical and diagnostic institutions in Germany and were isolated from clinical specimens. The studied strains are deposited at the Jena Microbial Resource Collection (JMRC). Table S1 provides a complete overview of the strains, including sources of isolation, JMRC strain collection numbers, and GenBank accession numbers.

### Molecular species identification

DNA extraction from *Fusarium* cultures and PCR amplification was performed as previously described (17). Oligonucleotides used for PCR amplification and sequencing are listed in Table S2. Sequences generated for species identification were deposited in GenBank. Accession numbers are listed in Table S1.

### *In vitro* antifungal susceptibility testing after EUCAST

The *in vitro* antifungal susceptibility testing was performed via broth microdilution technique according to the European Committee on Antimicrobial Susceptibility Testing (EUCAST) standard methodology (51). The following antifungals were tested: natamycin (Chemicalpoint, Deisenhofen, Germany); terbinafine (Chemicalpoint); olorofim (F2G, Ltd., Manchester, UK); manogepix (Basilea, Allschwil, Switzerland); rezafungin (Mundipharma, Frankfurt am Main, Germany); and ibrexafungerp (GSK, London, UK). Microplates were prepared by batch and stored at −80°C for use within 6 months. Each row contained one antifungal, which was twofold serially diluted starting from well 1 with the highest concentration to achieve the following test concentrations: natamycin and terbinafine (32 to 0.06 mg/L); and olorofim, manogepix, rezafungin, and ibrexafungerp (8 to 0.016 mg/L). *Fusarium* isolates were grown on malt extract agar plates for 3 to 5 days at 30°C. Spore suspensions were prepared in sterile dest. H_2_O with 0.1% Tween and counted with a hemocytometer. The final inoculum per well was 4 × 10^5^ spores/mL. Plates were incubated for 2 days at 35°C. MIC endpoints were determined visually using a mirror for natamycin, terbinafine, and olorofim. For manogepix, rezafungin, and ibrexafungerp, the MEC was determined by reading the microplates with the aid of an inverted microscope. For olorofim susceptibility, absorbance was further measured with a Tecan Infinite M Nano^+^ (Tecan, Switzerland) at 530 nm at *t*0 and after 2 days because the visual readout for olorofim activity against distinct *Fusarium* species appeared difficult to read due to slowly decreasing turbidity. Visual MIC using a mirror was determined analogously to azole activity; MEC using an inverted microscope was also determined analogously to echinocandin activity. The *Aspergillus fumigatus* strain ATCC 204305 was used as a control for antifungal activity. For the calculation of geometric mean MICs/MECs, off-scale values were raised to the next higher concentration.

## SUPPLEMENTAL MATERIAL

Fig. S1 aac.01913-24-s0001.tiff Exemplary images of *Fusarium* spp. exposed to manogepix.

Fig. S2 aac.01913-24-s0002.tiff Exemplary images of *Fusarium veterinarium* exposed to olorofim.

Fig. S3 aac.01913-24-s0003.tiff Comparison of *Fusarium* olorofim susceptibility using different readout methods.

Supplemental material aac.01913-24-s0004.docx Supplemental figure captions.

Supplemental tables aac.01913-24-s0005.xlsx Tables S1 and S2.

ASM does not own the copyrights to Supplemental Material that may be linked to, or accessed through, an article. The authors have granted ASM a non-exclusive, world-wide license to publish the Supplemental Material files. Please contact the corresponding author directly for reuse.
