# Supplementary figures and images for: In vitro activity of novel antifungals, natamycin, and terbinafine against Fusarium
Source: Antimicrob Agents Chemother. 2025 May 15;69(6):e01913-24. doi: 10.1128/aac.01913-24 (PMC12135539; doi:10.1128/aac.01913-24)

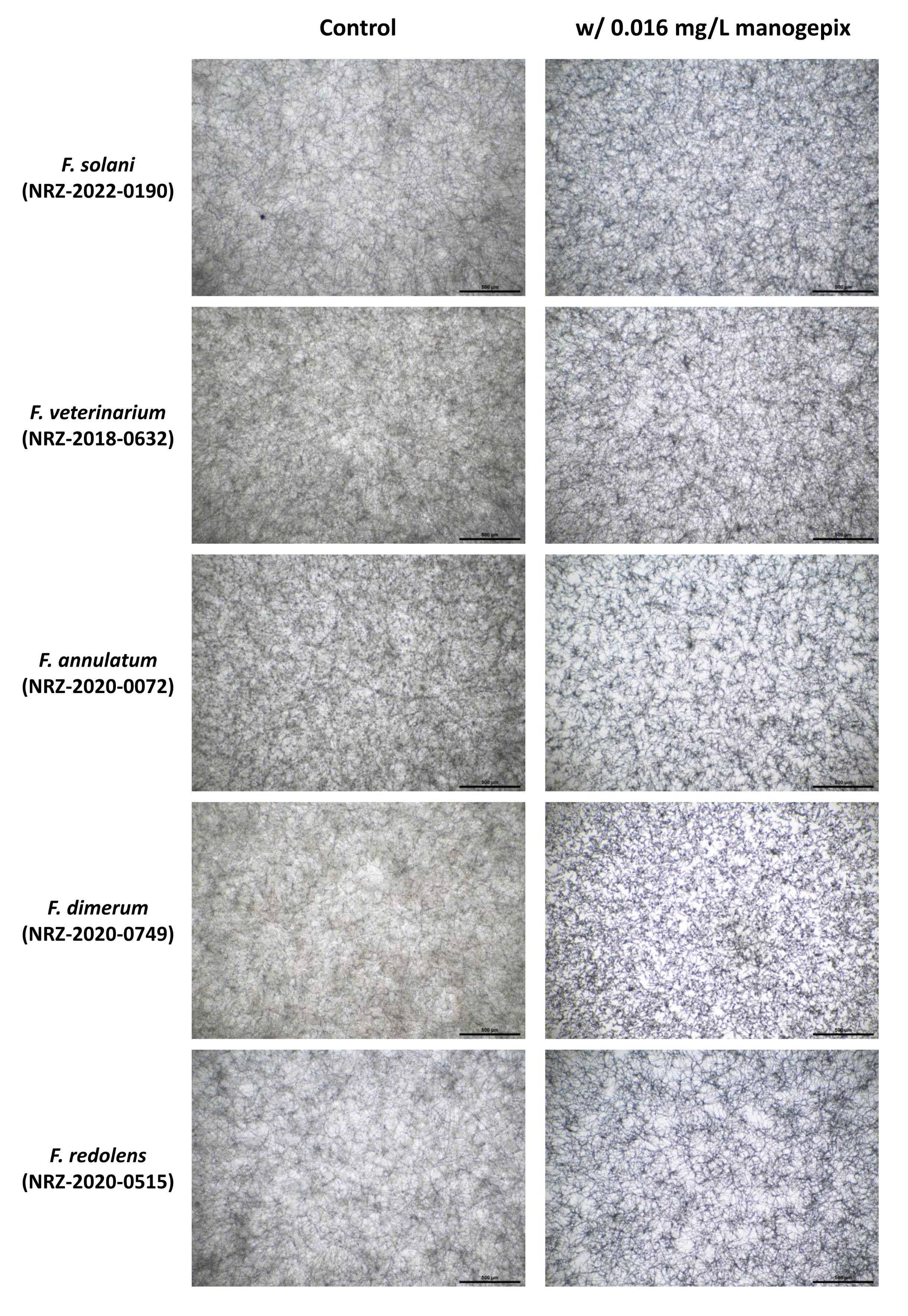

Supplement: Fig. S1 — Exemplary images of Fusarium spp. exposed to manogepix. [file aac.01913-24-s0001.tiff]

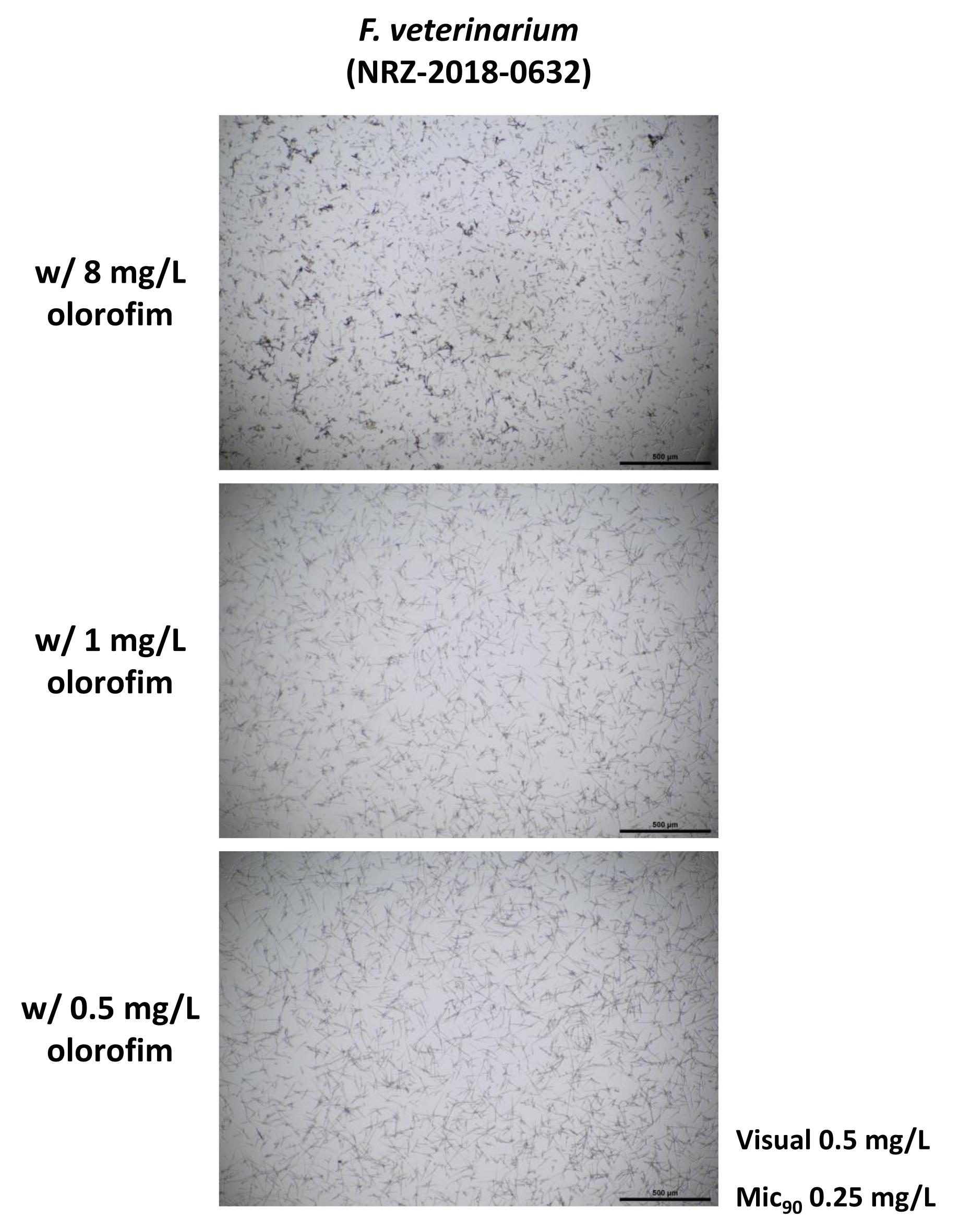

Supplement: Fig. S2 — Exemplary images of Fusarium veterinarium exposed to olorofim. [file aac.01913-24-s0002.tiff]

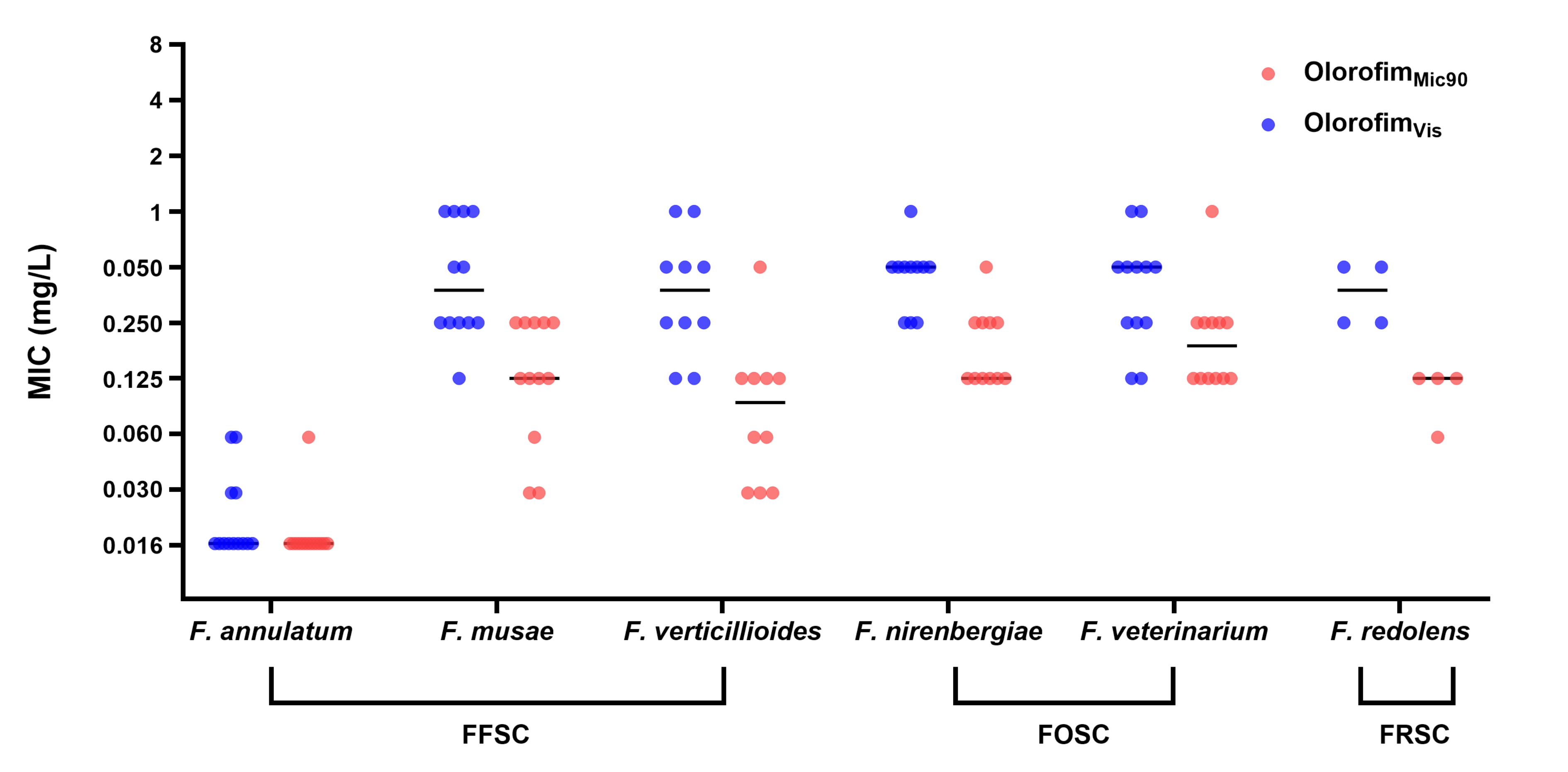

Supplement: Fig. S3 — Comparison of Fusarium olorofim susceptibility using different readout methods. [file aac.01913-24-s0003.tiff]
