# Supplementary material for: In vitro activity of novel antifungals, natamycin, and terbinafine against Fusarium
Source: Antimicrob Agents Chemother. 2025 May 15;69(6):e01913-24. doi: 10.1128/aac.01913-24 (PMC12135539; doi:10.1128/aac.01913-24)
Supplement: Supplemental material — Supplemental figure captions. [file aac.01913-24-s0004.docx]

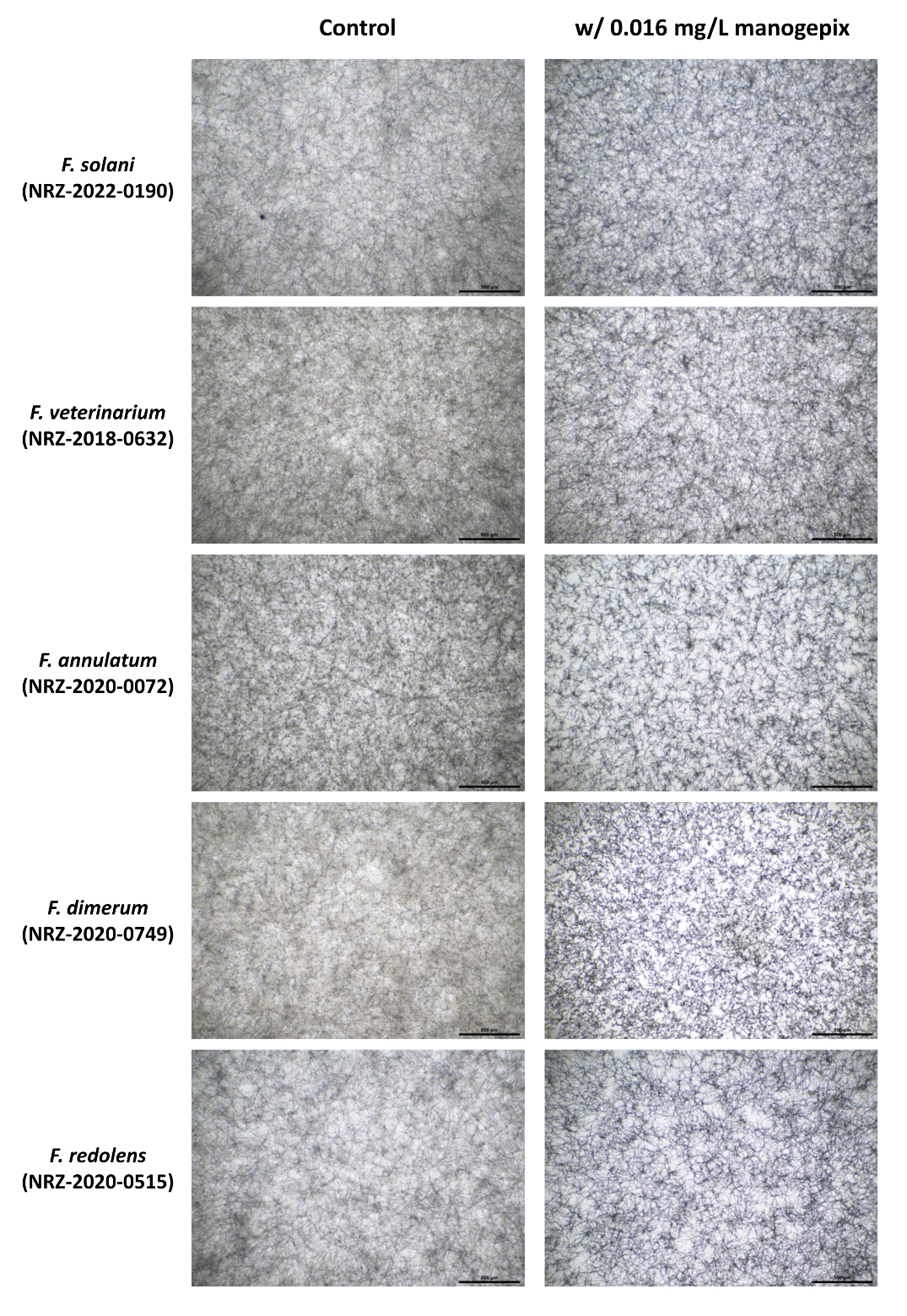


**Fig. S1: Exemplary images of *Fusarium spp*. exposed to manogepix**

Exemplary microscopy images of selected *Fusarium* isolates from EUCAST microdilution. Images were taken after 2 days incubation at 35°C from control conditions and incubation with 0.016 mg/L manogepix.


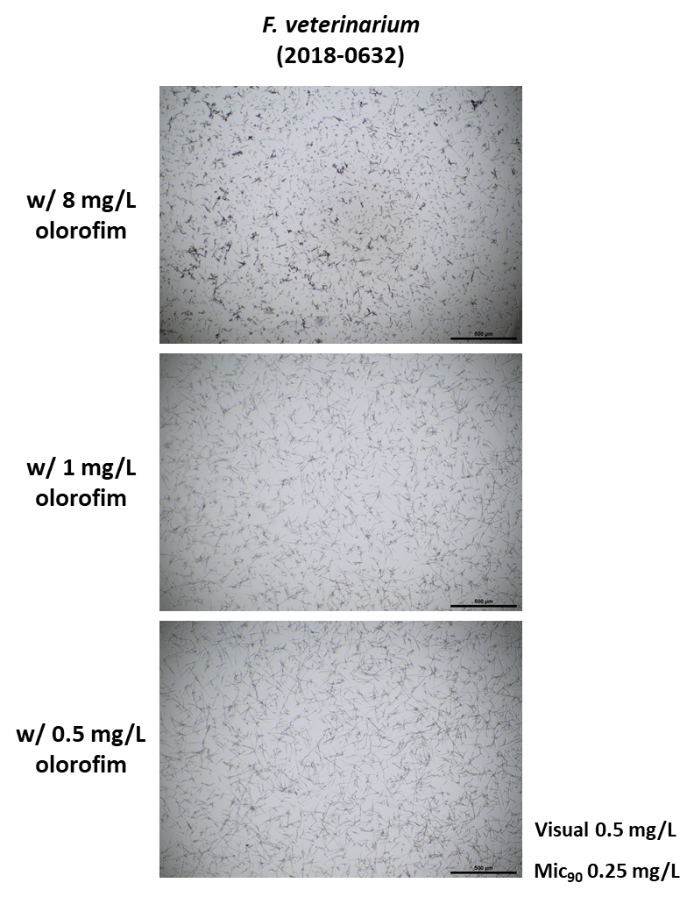


**Fig. S2: Exemplary images of *Fusarium veterinarium* exposed to olorofim**

Exemplary microscopy images from *F. veterinarium* isolate NRZ-2018-0632 after exposure to olorofim in EUCAST microdilution (2 days incubation at 35°C).


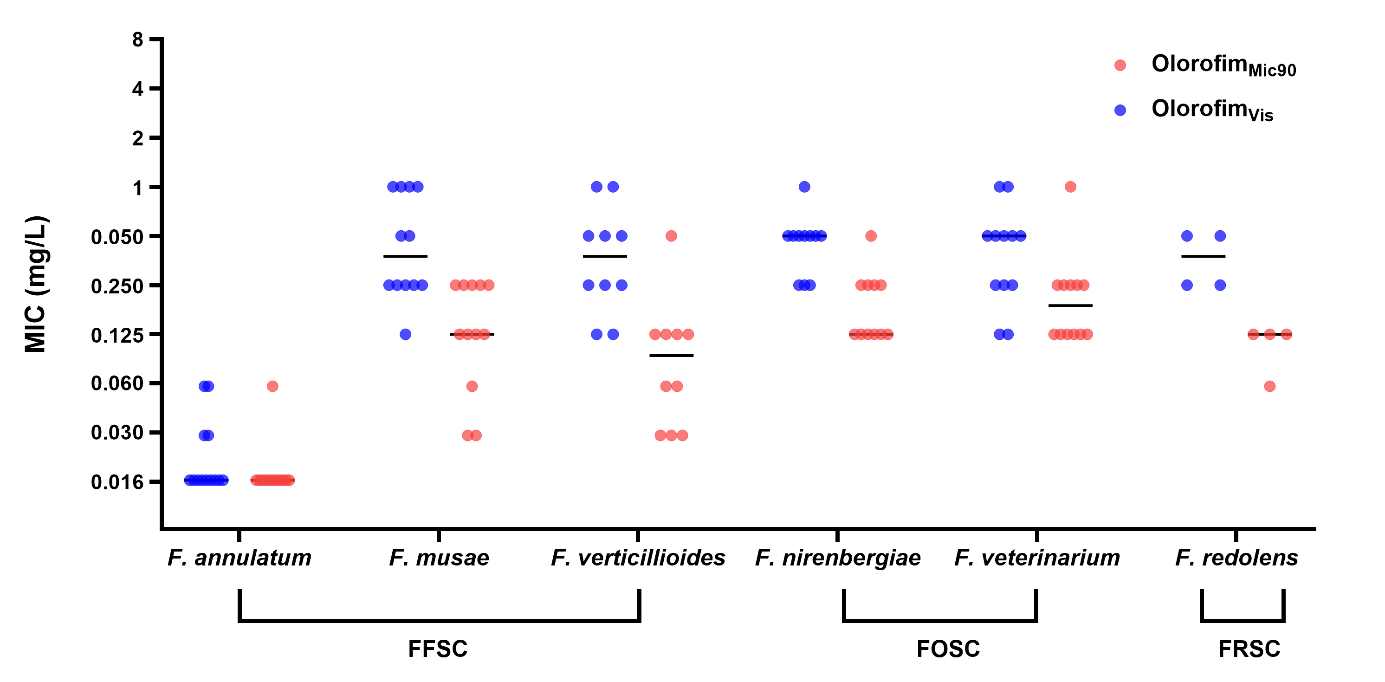


**Fig. S3: Comparison of *Fusarium* olorofim susceptibility using different readout methods**

Overview of olorofim MICs for selected *Fusarium* species assessed using either visual readout (Olorofim_Vis_) or 90% growth inhibition measured via spectrophotometer (Olorofim_Mic90_).
